# Editorial: Hormonal Control of Important Agronomic Traits

**DOI:** 10.3389/fpls.2018.01504

**Published:** 2018-10-17

**Authors:** Chi-Kuang Wen, Yunde Zhao, Yong-Ling Ruan

**Affiliations:** ^1^National Key Laboratory of Plant Molecular Genetics, CAS Center for Excellence in Molecular Plant Sciences, Shanghai Institute of Plant Physiology and Ecology, Chinese Academy of Sciences, Shanghai, China; ^2^Section of Cell and Developmental Biology, University of California, San Diego, La Jolla, CA, United States; ^3^School of Environmental & Life Sciences, University of Newcastle, Callaghan, NSW, Australia

**Keywords:** plant hormones, light signaling, auxin, ethylene, ABA, brassinosteroids, strigolactones, agronomic traits

From early 1950s to late 1960s, crop productivity was greatly increased by the adoption of the integrated agricultural practice involving application of chemical fertilizers, pesticides, irrigation, and high-yield variety crops with weakened gibberellin pathways. The high-intensity agricultural practice and the prevalent use of high-yield crop varieties, on the other hand, may bring profound harm to our environment and greatly impact agrodiversity (Pingali, 2012). With a rapid increase of the global population and depletion of natural resources, sustainable agricultural practice becomes critical for food security and environmental conservation. Plant hormones and their signaling networks are involved in almost all aspects of plant growth and development, and this Frontiers research topic covers a breadth of studies on hormonal controls of plant traits and biological activities, which may potentially be applied to improve agronomic traits, agricultural diversity, and sustainability.

The environmental signal light and plant hormones coordinately regulate many aspects of plant growth and development. One important light response is shade avoidance. Plants grown under canopy can sense the shade by detecting reduced red/far red light ratio and are able to adjust growth and development accordingly (Smith, 2000; Martínez-García et al., 2014). Chlorophyll biosynthesis upon exposure of etiolated plant tissues to light marks the initiation of photoautotrophic growth, and its degradation may facilitate nutrient reallocation upon senescence and fruit ripening. Three articles, by Yang and Li, Liu et al., and Zhu et al., respectively, reviewed regulations of shade response, chlorophyll biosynthesis, and degradation by light in concert with plant hormones. These articles addressed the integration of the external light signal with the internal hormone signaling network in the control of morphogenesis and other important biological processes such as stem elongation and flowering time.

Hormonal responses may interweave with nitrogen-use efficiency. In this context, the mutation of DELLA proteins involved in the green revolution rice varieties results in the inhibition of gibberellin responses and nitrogen assimilation efficiency (Li et al., 2018). Nitrate is a major nitrogen source for most land plants. Here, Guan reviewed the nitrate transport, signaling, and the interplay of nitrate with the biosynthesis and signaling of plant hormones. The intensive application of nitrogen fertilizers in modern agricultural practice may thus alter plant hormone homeostasis and responses, and the interplay may be a new research topic important for sustainable agriculture and environmental conservation.

Auxin plays pivotal roles in almost every major developmental process throughout the life cycle of a plant. Malka and Cheng reviewed the relationship between auxin biosynthesis and the production of the plant secondary metabolite glucosinolates (GLS). They proposed that auxin homeostasis might be modulated by GLS metabolism via the interaction of the biosynthetic pathways of indole glucosinolates and auxin. Plant cells can be programed to differentiate into root cells from explants and calli, namely direct and indirect *de novo* root regeneration, respectively. Yu et al. proposed that the steps for cell fate transition require auxin and are very similar between the two types of root regeneration.

ABA regulates several aspects of root development. Wang et al. reported that the promotion of rice root hair elongation by ABA is achieved through the positive control of auxin biosynthesis and transport. A related finding was that an elevated miR156 level is required for the auxin-induced adventitious root formation in *Malus xiaojinensis*.

Ethylene is a gaseous plant hormone, and ethylene response is regulated at both the biosynthesis and signaling levels. As the immediate ethylene biosynthesis precursor, ACC levels are tightly associated with ethylene production. Vanderstraeten and Van Der Straeten assessed the regulations of ACC pool and discussed a novel field of study on ACC as a potential signal molecule independent of ethylene action. The stability of Type I and Type II ACC Synthases (ACSs) is dependent on the phosphorylation status of the C-terminal region (Xu and Zhang, 2015). The Type III ACS7 does not have the C-terminal extension and its degradation is mediated by the RING-type E3 ligase XBAT32 (Lyzenga et al., 2012). Sun et al. reported involvement of the N-terminal 14 amino acid residues in ACS7 degradation, which is negatively regulated by leaf senescence, revealing a regulation of ACS7 stability by the senescence signal. As an important gaseous plant hormone, aspects of ethylene-related agronomic traits of regular rice varieties were reviewed by Yin et al. The nuclear localization signal of the ER protein EIN2 is essential to ethylene signaling activation (Zhang and Wen, 2015). Interestingly, Kessenbrock et al. reported an interaction of the tomato ethylene receptors LeETR4 and Never-ripe (Nr) with the synthetic peptide derived from EIN2 NLS sequence. The peptide may have agricultural value serving as a ripening inhibitor. The finding that the NLS-peptide affects ethylene responses may also shed light on how ethylene signaling is regulated.

Crop architecture is a key yield-determining trait (Wang et al., 2018), and the green revolution was directly resulted from using semi-dwarf crop varieties, which decreased lodging-related yield loss (Li et al., 2018). The plant hormones brassinosteroids (BRs) and strigolactones play prominent roles in shaping plant architecture. The study by Hu et al. revealed that elevated levels of gibberellins and BRs contribute to maize heterosis for plant height. The rice plant architecture can be regulated by the transcription factor OPF1, which interacts with the GRAS protein DLT in response to BRs (Xiao et al.). Strigolactones promote branching (tilling) of rice plants, and Hu et al. unveiled the underlying mechanism of a novel feedback regulation. The perception of strigolactones by the enzyme-receptor protein DWARF14 leads to its ubiquitination at Lys280 residue and subsequent degradation. Nie et al. reported the improvement of several major agronomic traits in tomato by overexpressing the BR receptor *SlBRI1*.

Research tools are continuously innovated to drive new discoveries. Yang et al. developed a series of auxin biosensors that facilitate studies on dynamic regulation of auxin responses throughout development of the rice plant. The GSHR web server (http://bioinfo.sibs.ac.cn/GSHR/) by Ran et al. facilitates the mining of biologically significant data for understanding plant hormone responses via cross-platform comparisons. Song et al. reported single-molecule fluorescence methods that can be widely applied to study the interaction kinetics of signaling components of a signal transduction pathway, and a competitive association of the BR signaling components BKI1 and BES1 with 14-3-3κ was exemplified.

The modern plant biology covers a wide variety of studies, such as genomics, transcriptomics, proteomics, genetics, and molecular biology. Its progress is often facilitated by the development of innovated tools. Plant growth and development are fine-tuned and dynamically regulated at many levels of hormonal control and interactions. Integration of the various fields of modern plant biology studies and understanding the complexity, plasticity, and the underlying mechanisms will aid diversifying agricultural practice and breeding to improve sustainable agriculture, agrodiversity, and environment.

## Author contributions

C-KW wrote the manuscript. YZ and Y-LR made revisions.

### Conflict of interest statement

The authors declare that the research was conducted in the absence of any commercial or financial relationships that could be construed as a potential conflict of interest.
